# Predicting a double mutant in the twilight zone of low homology modeling for the skeletal muscle voltage-gated sodium channel subunit beta-1 (Na_v_1.4 β1)

**DOI:** 10.1016/j.csbj.2015.03.005

**Published:** 2015-03-27

**Authors:** Thomas Scior, Bertin Paiz-Candia, Ángel A. Islas, Alfredo Sánchez-Solano, Lourdes Millan-Perez Peña, Claudia Mancilla-Simbro, Eduardo M. Salinas-Stefanon

**Affiliations:** a Facultad de Ciencias Químicas, Universidad Autónoma de Puebla, Puebla, Mexico; b Laboratorio de Biofísica, Instituto de Fisiología, Universidad Autónoma de Puebla, Puebla, Mexico; c Centro de Química, Instituto de Ciencias, Universidad Autónoma de Puebla, Puebla, Mexico

**Keywords:** MD-2, myeloid differentiation factor 2 (MD-2), SDM, site-directed mutagenesis, Na_v_1.4, skeletal muscle voltage-gated sodium channel, TLR4, Toll-like receptor type 4, Trk, tyrosine receptor., Ig-like, CDR1, MD-2, Patch-clamp, Site-directed mutagenesis, Analogy modeling

## Abstract

The molecular structure modeling of the β1 subunit of the skeletal muscle voltage-gated sodium channel (Na_v_1.4) was carried out in the twilight zone of very low homology. Structural significance can *per se* be confounded with random sequence similarities. Hence, we combined (i) not automated computational modeling of weakly homologous 3D templates, some with interfaces to analogous structures to the pore-bearing Na_v_1.4 α subunit with (ii) site-directed mutagenesis (SDM), as well as (iii) electrophysiological experiments to study the structure and function of the β1 subunit. Despite the distant phylogenic relationships, we found a 3D-template to identify two adjacent amino acids leading to the long-awaited loss of function (inactivation) of Na_v_1.4 channels. This mutant type (T109A, N110A, herein called TANA) was expressed and tested on cells of hamster ovary (CHO). The present electrophysiological results showed that the double alanine substitution TANA disrupted channel inactivation as if the β1 subunit would not be in complex with the α subunit. Exhaustive and unbiased sampling of “all β proteins” (Ig-like, Ig) resulted in a plethora of 3D templates which were compared to the target secondary structure prediction. The location of TANA was made possible thanks to another “all β protein” structure in complex with an irreversible bound protein as well as a reversible protein–protein interface (our “Rosetta Stone” effect). This finding coincides with our electrophysiological data (disrupted β1-like voltage dependence) and it is safe to utter that the Na_v_1.4 α/β1 interface is likely to be of reversible nature.

## 1 Introduction

### 1.1 The function and structure of Na^+^ channels

Ion channels are a ubiquitous class of membrane-spanning proteins. They accomplish electrochemical functions and specifically regulate ion movements (Na^+^, K^+^, Ca^++^cations or Cl^−^ anions) through their gating mechanism, understood as the transition between open active, inactive and closed states. A typical channel is a multimeric protein complex. It is assembled from a pore-forming α subunit that is often assisted by other subunits labeled β, γ, δ, etc. [1] Mammalian Na^+^ channels are heterotrimers, composed of one central α subunit of four variable repeat units or domains (DI to DIV) and two or more auxiliary β subunits. Nine α isoforms and 4 β isoforms have been described for this class [2]. For many ion channels (Na^+^, Ca^++^, GABA, and NMDA) subunit cooperativity is paralleled by small molecule modulation through interaction sites other than the pore region with its outer and inner vestibules. Such ligand binding sites are often referred to as allosteric, modulatory or regulatory [3].

### 1.2 The Na^+^ channel β1 subunit (Na_v_β1)

Na^+^ channel β subunits were functionally characterized as channel gating modulators and channel protein expression regulators at the plasma membrane level and were structurally identified as “cell adhesion molecules” [4,5]. The β subunit modulation confers differential activity depending on the channel isoform and tissue type where the protein complex is expressed. The primary sequence of the sodium channel β subunit (Na_v_β1) is the same for all α subunit isoforms [6]. The presence of Na_v_β1 is a necessary but not sufficient prerequisite to modulate channel activity. The extracellular domain of β1 is necessary and sufficient to modulate the channel gating of α subunit isoforms Na_v_1.2 and Na_v_1.4; this subunit accelerates channel inactivation and recovery from inactivation [7–9]. In more explicit terms the Na^+^ channel gating, in presence of the β1 subunit, changes from slow to fast mode at different extents in practically every isoform except in Na_v_1.5, which predominates in cardiac myocytes and exhibits fast gating on its own [10]. In stark contrast, the skeletal muscle isoform Na_v_1.4 requires the co-expression of β1 to reconstitute the native fast Na^+^ currents [4,11].

### 1.3 Na^+^ channel α and β subunit models

At present no crystal structure of a full α subunit eukaryote Na^+^ channel has been published. Currently the best template to model a mammalian α subunit constitutes the bacterial channel Na_v_Ab (PDB codes: 4EKW [12] and 3RVY [13]) which has a 33% identity (E value of 2 e^− 13^) with respect to rNa_v_1.4 isoform (100%). Homology is a prerequisite for reliable 3D template modeling of target proteins with unknown structure. An intriguing question for ion channel researchers over the recent years has been how to gain insight into the cooperativity between α and β1 subunits of the Na^+^ channels despite the absence of crystallographic data.

Heterotetrameric voltage-gated Ca^++^ and Na^+^ channels α subunits are thought to be homologous, sharing a common ancestral K^+^ channel and being originated by gene duplication separately, or Na^+^ channels having evolved from Ca^++^ channels. This reasoning comes from the interesting fact that the four domains DI to DIV of the Na^+^ channels are more similar to the corresponding four repeats of Ca^++^ channels than resemblance between each other [14,15]. Each domain in both ion channels possesses six transmembrane segments (S1 to S6) and the central pore region is constituted by a S5-p-S6 fold unit, while transmembrane helix S4 is considered the voltage sensor giving response to the electrical depolarization stimuli and thusly initiating the channel opening for ion flux [2,16].

The naming convention of accessory subunits among these channels, however, is inconsistent, for instance the β subunit of the voltage-gated Ca^++^ channel (Ca_v_), which has been crystallized in complex with its Ca_v_α interface (PDB code: 1T0J [17]) is located on the intracellular side and it has a 13.4% identity to Na_v_β1 but unlike the latter, the former belongs to the P-loop containing nucleotide triphosphate hydrolase superfamily. Conversely, the Ca_v_α2δ subunit resembles more our target Na_v_β1. Although named “delta” it embraces a domain with the same fold unit as target Na_v_β1. Moreover it shows a two-peptide complex linked by two disulfide bridges [18]. Thus we dismissed the Ca_v_ templates for Na_v_β1 modeling. No wonder, former homology models of target Na_v_ channels have been based on another oligomeric channel type, namely the voltage-gated potassium channel (K_v_). Although it possesses a β subunit (K_v_β, KCNAB family) which modulates channel gating, K_v_ differs due to its function as an oxidoreductase enzyme as well as its location in complex with α cytoplasmic segments (PDB code: 1QRQ [19]).

Surprisingly, Na_v_β1 subunit also modulates members of a K^+^ channel subfamily. Mutational studies of Shaker K^+^ channels have assisted in the generation of a computational model of the K_v_1.2–Na_v_β1 interface [20]. Today, much better resolved crystal structures of voltage-gated K^+^ channels have lend detailed insight into their topologies (PDB codes: 3LUT [21]). Unlike eukaryotic α K_v_ channels (composed of 4 subunit chains), the Na_v_ channels share the same topology of four variable transmembrane domains, loops and voltage sensor on a single subunit chain with Ca_v_ channels [22].

The advent of crystal structures of full bacterial voltage-gated Na^+^ channels such as NaChBac and Na_v_Ab (PDB code: 4DXW [23] and 3RVY [13]) has allowed the modeling of full heteromeric α eukaryote subunits by homology [24,25]. Prior to the advent of bacterial Na^+^ templates extant models had been generated from K^+^ channel templates such as the bacterial MthK channel. They included only pore-forming domains. At that time pore width and other geometrical data for modeling were inferred from channel blocker ligand studies [25–28]. Up to now, more structural insight is in need, e.g. the loop lengths and overall geometries of the highly variable segments or the outer and inner vestibules. At the end of the present study about predicting the interacting residues, the crystal structures of Na_v_β3 and Na_v_β4 were published (PDB codes: 4L1D [29]; 4MZ2 [30]). Yet, due to insufficient data the molecular mechanism of interaction between Na_v_α and β1 subunits remains to be elucidated at an atomic level.

Homology models of the α subunit for the wild type isoforms Na_v_1.4 and β1 subunit had already been used in our laboratory to assist the experimental work [11,28,31]. Here we combined computed protein structure prediction, site-directed mutagenesis (SDM) and electrophysiological studies to investigate the possible function of relevant amino acids, involved in the inactivation process. Our results showed that two adjacent residues (threonine T109 and asparagine, N110) had a critical role in the inactivation process. When we mutated threonine 109 and asparagine 110 to alanine (T → A; N → A, called TANA), the kinetic process of inactivation was affected generating a general loss of function.

## 2 Materials and methods

### 2.1 Searching 3D template for the generation of the target subunit β1

Since the crystal structure of the mammalian Na^+^ channel subunit β1 (Na_v_β1) has not been elucidated the 3D target model was generated from its primary sequence data from UniProt [4,32] and a related 3D template (PDB database [33]). To this end we searched for homologous crystal templates to generate the target 3D model among the known PDB database entries by FASTA and BLAST [34,35]. All templates belonged to the general class of “all β protein” structures, in particular the immunoglobulin superfamily with the so-called immunoglobulin-like fold motif (Ig-like domain). As a data subset we collected all 3D templates of an Ig-like protein in complex with any other protein, regardless of its relatedness (homology) to the Na_v_α subunit. In this way, the study was based on a combined homology and analogy approach. On the one hand, homology was used for the Na_v_β1 model generation, while on the other hand, analogous interfaces were studied which were formed between cell adhesion proteins of the “all β protein” class and any other protein type whether or not it was found homologous or not (analogous) to the Na_v_α subunit. Of note, the denomination “beta” for the channel's subunit β protein coincides with the “all β protein” class, but without intention to label a “beta fold” motif as such. This becomes evident in the case of calcium channel β subunits which do not belong to the “all β protein” class. Here Greek letters merely label the subunits (proteins in complex).

### 2.2 Alignments of sequences and secondary structure determination

It is also noteworthy to state, Chimera [36] or Swiss PDB Viewer [37] could not resolve automated structure alignments in all cases. On occasion, it became necessary to help out by manual superposition (Vega ZZ [38]). To this end, some atoms were selected in the N-terminal and C-terminal regions, others in the loop regions, namely the turns between strands A to B and E to F.

Automated multiple sequence alignments (MSA) and sequence identity determinations were carried out with web-based programs and software package tools (Clustal W, Chimera) [39,36]. The secondary structure for the target sequence was estimated as a consensus (overlay) of results by prediction tools NPSA and JPRED [40,41]. In addition, hair pin loops were assessed, i.e. type II turns with the general pattern “XG”, where X is any (one) amino acid [42]. The result was compared to the secondary structures of known crystal structures with Ig-like domains [43].

### 2.3 Manual subunit β1 modeling

The present model was generated using SCRWL [44] and Vega ZZ [38] for manual threading of the target sequence (UniProt [4,32]) across the chosen template structure – in contrast to our earlier multiple template models generated with I-TASSER [45,11] and Modeller [46]. In particular, I-TASSER – an acronym for *iterative threading assembly refinement server* – required the target sequence of the unknown structure in FASTA format as input data. The program automatically launches a 3D-template search (psi-Blast) and reports the homologous proteins from the protein data bank (PDB [33]), assisted by their sequence profiles (psi-pred), while the query sequence is threaded through a collection of possible 3D templates (multiple template construction) [47]. Our topological analyses were documented by web-based tool Topo 2D/TMRPres2D [48]. Moreover, Vega ZZ was served as a general purpose modeling tool [38].

A step-wise description of the combined homology/analogy modeling approach is given in the following Results section.

### 2.4 Chinese Hamster Ovary (CHO) cell co-transfection

CHO-K1 cells were transiently transfected with rat Na_v_1.4 cDNA (UniProt accession number P15390) which was cloned into the pGW1H (1 μg) and with cDNA of either native or mutated rNa_v_β1 (2.5 μg each). Then cDNA was mixed with Lipofect AMINE Plus reagent (Gibco, Invitrogen). CHO-K1 cells were maintained in Dulbecco's modified Eagle's medium (Invitrogen) supplemented with 6% fetal bovine serum (Gibco, Invitrogen), 0.1 mM hypoxanthine, and 0.01 mM thymidine at 37 °C in a 5% CO_2_ humidified incubator. The transfected cells were given fresh Dulbecco's modified Eagle's medium containing 1000 U penicillin, 0.1 mg streptomycin + 0.25 μg of amphotericin B per ml, and were passaged at 2- to 3-day intervals with a brief trypsin–EDTA treatment. The cells were dissociated and seeded onto glass coverslips (12-mm diameter; Fisher Scientific, Pittsburgh, PA, USA) in a 35-mm dish 1 day before use. For electrophysiological experiments, coverslips with attached cells were transferred to a recording chamber (RC-13; Warner Instruments, Hamden, CT, USA). The chamber was superfused at a rate of 0.5 ml min^− 1^ with normal external solution at 36 ± 1 °C.

### 2.5 Site-directed mutagenesis and electrophysiology

Briefly, alanine substitutions in positions 109 and 110 were introduced in the rNavβ1 construct (Scnb1: Q00954) and cloned into a pGEMHE new vector with a single pair of mutagenic primers. Standard procedures and electrophysiology protocols were performed and applied as previously described [49]. Values are reported as the mean ± SEM. Statistical comparisons between two mean values were conducted by the unpaired Student's *t*-test. Graphs were built and fitted using Sigmaplot 11.0 (SPSS, Inc., Chicago, IL, USA) and Origin 8.02 (Origin Lab Corp., Northampton, MA, USA).

### 2.6 Electrophysiological recordings and data analysis

The cells were allowed to stand for 5 min to facilitate precipitation and adhesion. They were then perfused with external solution from electronic valves remote operated by a programmable controller (Val-505®; CIDES Technology, Puebla, Mexico) to allow the exchange and perfusion of the different solutions used. All experiments were performed at 36 ± 1 °C, which was regulated by a bipolar temperature controller (Medical Systems Corporation, Boston, MA, USA).

## 3 Results

In the following we lay out the technical procedure in seven steps – all of which were paramount to identify relevant amino acids on Na_v_β1 for the possible protein–protein interface between Na_v_1.4 α and β1 subunits. After the seven modeling steps we show the experimental results (Step 8).

### 3.1 Step 1: the target subunit β1

**Fig. 1:**
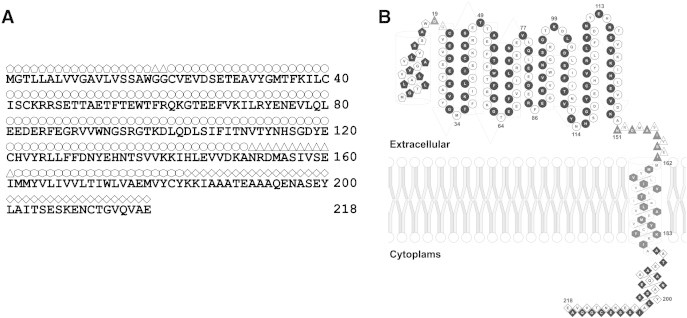
Schematic display of the main chain with its principal domains for the Na_v_β1 subunit. It documents the location of the major structural domains of Scn1b sodium channel voltage-gated subunit β type 1 *Rattus norvegicus* (accession code: Q00954) [4]. Shown are the sequence (A) and topology (B) which embraces the signal peptide (pentagons, length: 1–18), extracellular immunoglobulin domain (circles), transmembrane domain (hexagons), and intracellular domain (diamonds). The triangles symbolize the linker domain. Every second symbol is filled black or left white to mark the alternative neighbors.

The primary sequence of the rat Na^+^ channel subunit β1 (rNa_v_β1) was retrieved from the UniProt web service (accession code: Q00954 [4,32]). The extracellular domain was limited to 142 residues excluding the signal peptide (Fig. 1).

### 3.2 Step 2: inspection of known sodium channel structures

The initial search of suited 3D models of the voltage-gated ion channels left us with more open questions than reliable answers (Table 1). Although collecting structures of ion channels is a straightforward task, some implications fairly limit their practical use as 3D templates: (1) the types and (2) numbers of subunits (chains) of extant crystal structures (homo- or heterotetrameric repeat units), (3) the sequence similarities or (4) the specific residue variations responsible for ion selectivity in the repeat units, (5) the specific residues of the α/β1 interface situated in the structurally unknown loops or elsewhere, (6) in addition to residue changes due to phylogenetic distances among the published data for different species. None of the primary sequences of the ion channels (Table 1) showed homology to the heterotetrameric Na_v_α subunit [Clustal W [39]). With no reliable crystallographic data for the entire multimeric channel at hand we continued searching for suited 3D templates of the subunit Na_v_β1 alone.

### 3.3 Step 3: phylogeny of the target Na_v_β1 protein and its homology to 3D templates

According to the SCOP classification and annotation system, from all PDB entries (101,046 as of June 2014) over 48,700 structures fell into the top-level phylogenetic class of “all β proteins”. “All beta” means that the proteins are composed of β strands building up beta sheets. Within this lineage class, over 6500 structures belong to the folding motif “immunoglobulin-like β-sandwich”. The domain fairly resembles two bred buns like a sandwich (with nothing put in-between) for eating. Commonly, the domain possesses a Greek key architecture and seven or more β strands to form the two β sheets.

**Fig. 2:**
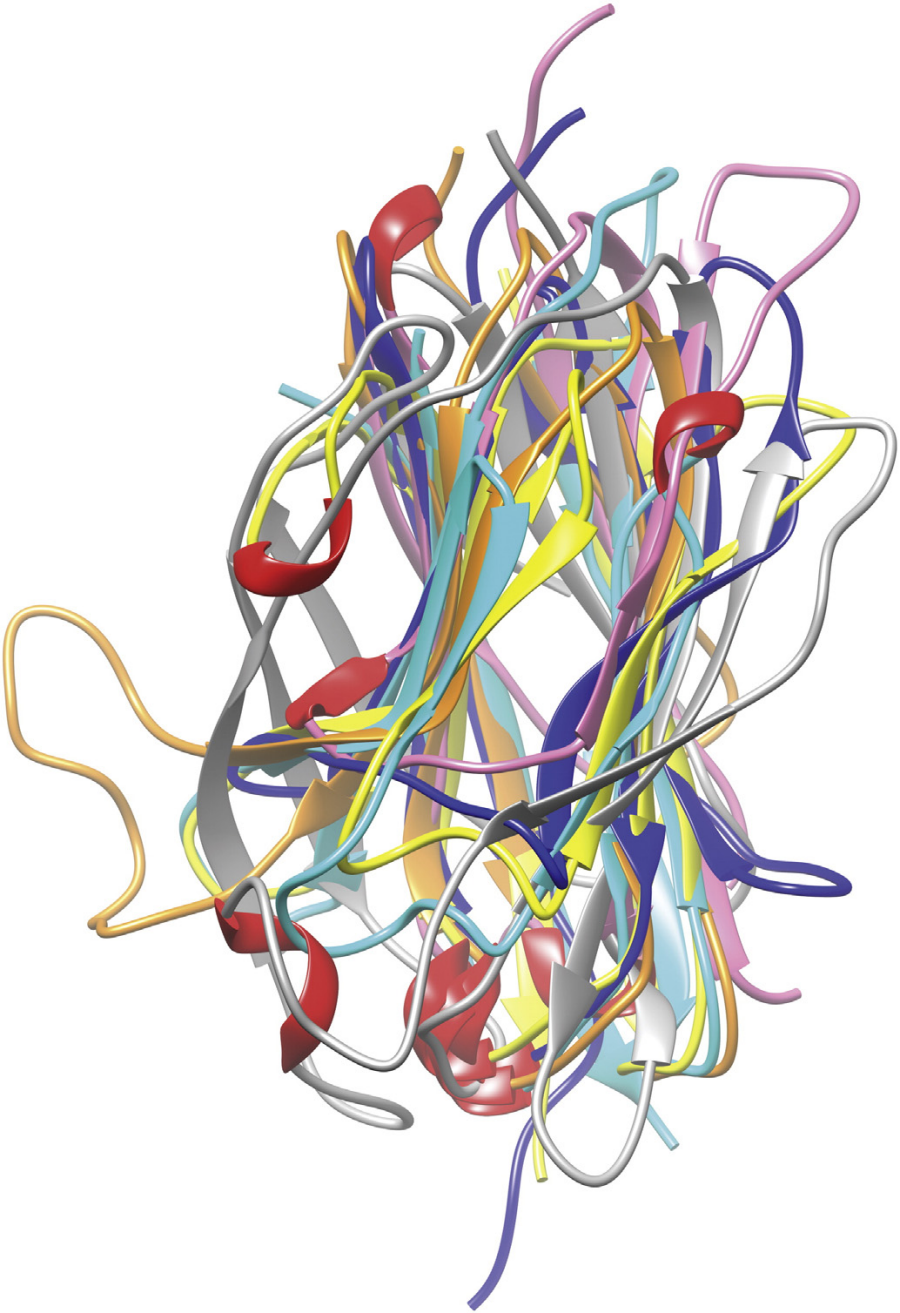
Display of Ig-like 3D templates in superposition. While the central strands follow a more regular pattern [42] the variable loops wrap up the common Ig-like fold at the surface. Protruding backbone lines (e.g. orange or gray tubes on either side) represent longer loops. Small 3_10_ helical segments (red) and beta strands of sheets (ribbons as arrows) are visible. In representation of all others, six superposed samples (cf. * in Table 2) were displayed and colored individually (dark blue 1HCF, pink 1JPS, light blue 1NEU, gray 1FXI, orange 3GRW, yellow 1KXQ) [59–61,72–74]. The N-terminal segments start on top (e.g. pink line), the C-terminal parts end toward the bottom (e.g. dark blue, light blue, yellow tubes). For interpretation of the references to color in this figure legend, the reader is referred to the web version of this article.

The PDB data base [32] was searched by FASTA and BLAST [34,35] for potential 3D templates of the target primary sequence of Na_v_β1. A great plethora of crystal structures with an Ig-like β sandwich fold exist. At this stage the study concluded with a trade-off between the sheer numbers of sequences versus a reduced sample set of 3D templates (Table 2) which were amenable to inspection and yet covering a wider range of structural variations (Fig. 2). Many Ig-like motifs are seen in extracellular parts of transmembrane proteins where they are involved in protein–protein interactions. Historically they were labeled by a collective name as “cell adhesion molecules” – although the name “cell protein adhesion molecule” would be more appropriate in our case [53–56]. Despite their different functions they all share a common fold unit, the Ig-like β sandwich structure. For instance, telokin (PDB code: 1FHG) [57] or the chaperone family (or heat shock proteins, HSP; PDB code: 3D2F [58]), the receptor tyrosine kinases (TrkA, B, C; PDB codes: 1WWA [53], 1WWB [53], 1WWC [53], 1HCF [59]); immunoglobulins (antibodies, PDB codes: 1JPS [60], 3GRW [61], 3KLD [62], 2GKI_A (rat) [63], 1KAC_B [64], and 3BKJ_H [65]); and antimicrobial protein tachycitin 1XT5_A [66], 1EAJ_A [67] or myelin protein zero 3OAI [68].

As can be judged by eyesight the sampled crystal structures show a wide range of loop variations (Fig. 2). In consequence, those crystal structures with a common fold unit were inspected which embraced a protein-liganded complexes regardless of the degree of overall sequence conservation (Table 3). Prior to the appearance of Na_v_β3 and β4 subunits (PDB codes: 4L1D [29]; 4MZ2 [30]) we used the hitherto known PDB entries as 3D templates (Table 2). At the time of modeling – during 2012 to 2013 – the homology between target primary sequence and potential 3D templates was found to be extremely weak (cf. * in Table 2). Below the threshold of around 30% for a 100 to 150 residue-sized domain, sequence alignments of template structures against the target sequence fell into the twilight zone of very low homology (Table 3).

### 3.4 Step 4: topology, sequence alignments of templates and threading of target Na_v_β1

At the time of modeling the sequence identities ranged between 11% and 23% for a residue length ranging from 101 to 384 – risking randomly aligned sequences as a direct result of confounding relevant with irrelevant residue positions by chance conservation (Table 3). Hence, the level of complexity was lowered to safer grounds of structural knowledge. To this end, two-dimensional topology diagrams of representative Ig-like β sandwich proteins were compared (cf. * in Table 2): BDNF/NT-3 growth factors receptor (PDB code: 1HCF_X [59]), antigen binding fragment Fab (PDB code: 1JPS_H [60]), Fibroblast growth factor receptor (PDB code: 3GRW_A [61]), Contactin-4 (PDB code: 3KLD_A [62]), Camelid Vhh antibody (PDB code: 1KXQ [72], Myelin protein P0 (PDB code: 1NEU_A [74]) and TLR4/MD-2 (PDB code: 3FXI_C [73]). In particular, their common structural elements – adjacent strands and loops, bonded or nonbonded neighbor residues, cysteine bridges, hydrogen-bond network, gaps in loops, β-turn-β and hair pin motifs – were analyzed (Fig. 2).

**Fig. 3:**
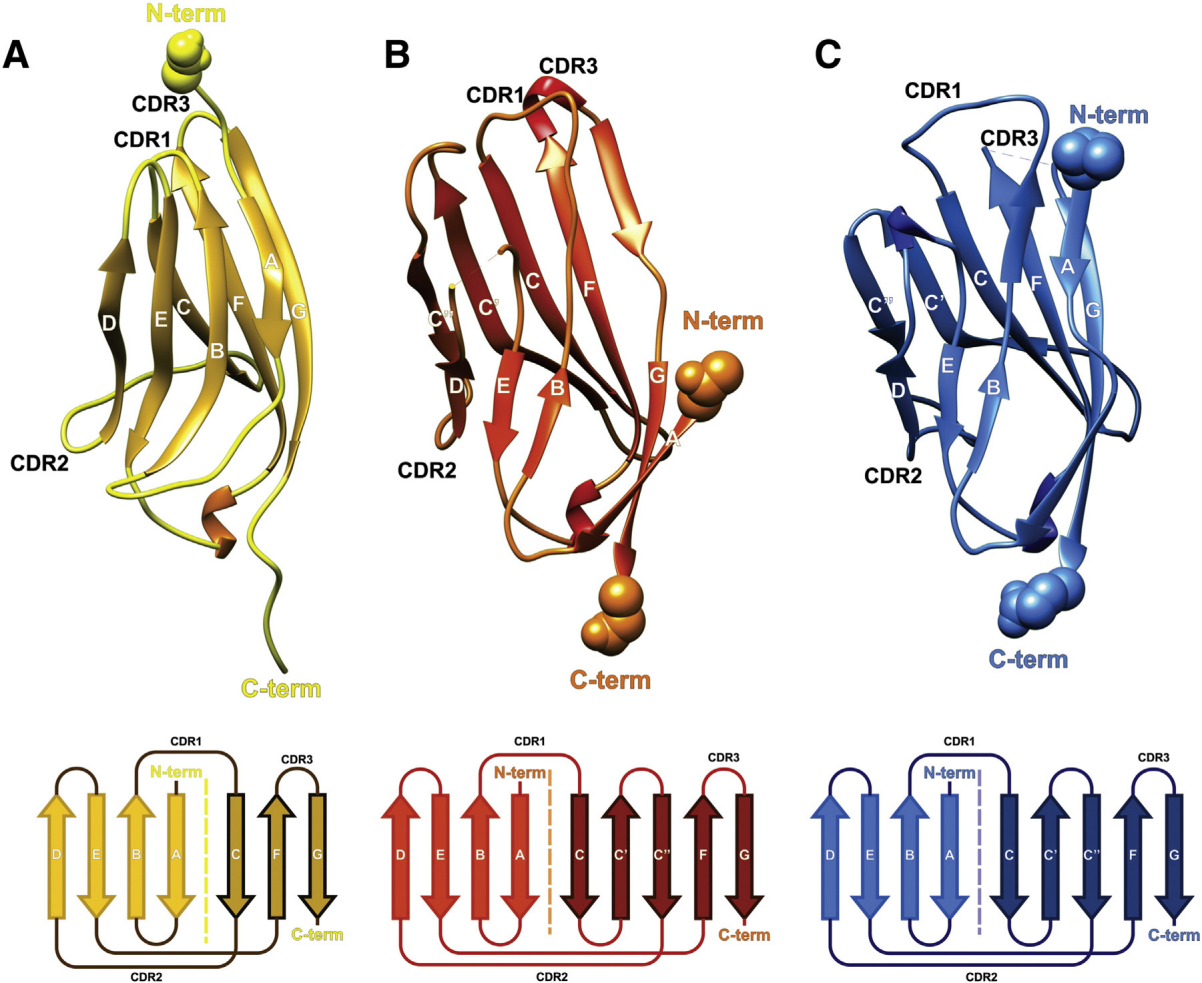
Display of protein models (first row) and topology schemes (bottom row) of the used template (panel A, 1HCF [59]) in comparison to Na_v_β4 (panel B, 4MZ2 [30]) and myelin ectodomain (panel C, 1NEU [74]). The β strands (arrow symbols) are labeled. Between β strands C and D (leftmost strand) a larger loop segment allows the formation of two extra β strands, labeled C′ and C″ (panels B and C) [42]. They were analyzed in the following step.

At the end of the present modeling study the crystal structures of Na_v_β3 and β4 were published (PDB codes: 4L1D [29]; 4MZ2 [30]). Now – during Spring 2015 – we carried out fully automated homology modeling of the target protein and compared the results to our manually generated model (Fig. 3) [59]. It was found that aiming at a higher overall id score was not necessary. A lower id score could include a better structural conservation in just the local hot spot(s). Despite its poor id score (19% in Table 3) our 3D template (PDB code: 1HCF) [59]) yet outperformed the higher scoring templates (PDB codes: 4L1D [29]; 4MZ2 [30]) for four good reasons: (i) allowing to pinpoint unexplored target surface areas in search of residues to be mutated (hot spots). (ii) Even templates with lower id score still conserve the Ig-like fold unit (β-sandwich core). (iii) Even with a higher id score above the twilight zone of homology, templates like 1NEU or Na_v_β3 and β4 do not present reliable loop coordinates. (iv) Albeit the overall structure of our 3D template had large variation to show in the loop parts – and therein it was not worse than any other template – the lengths, distances or twists of its fold geometry closely resembled that of the two crystal structures of β3 and β4 subunits: strand A–turn–strand B, strand C–(initial part of longer) loop–strand D, strand E–turn–strand F, or strand F–turn–strand G.

**Fig. 4:**
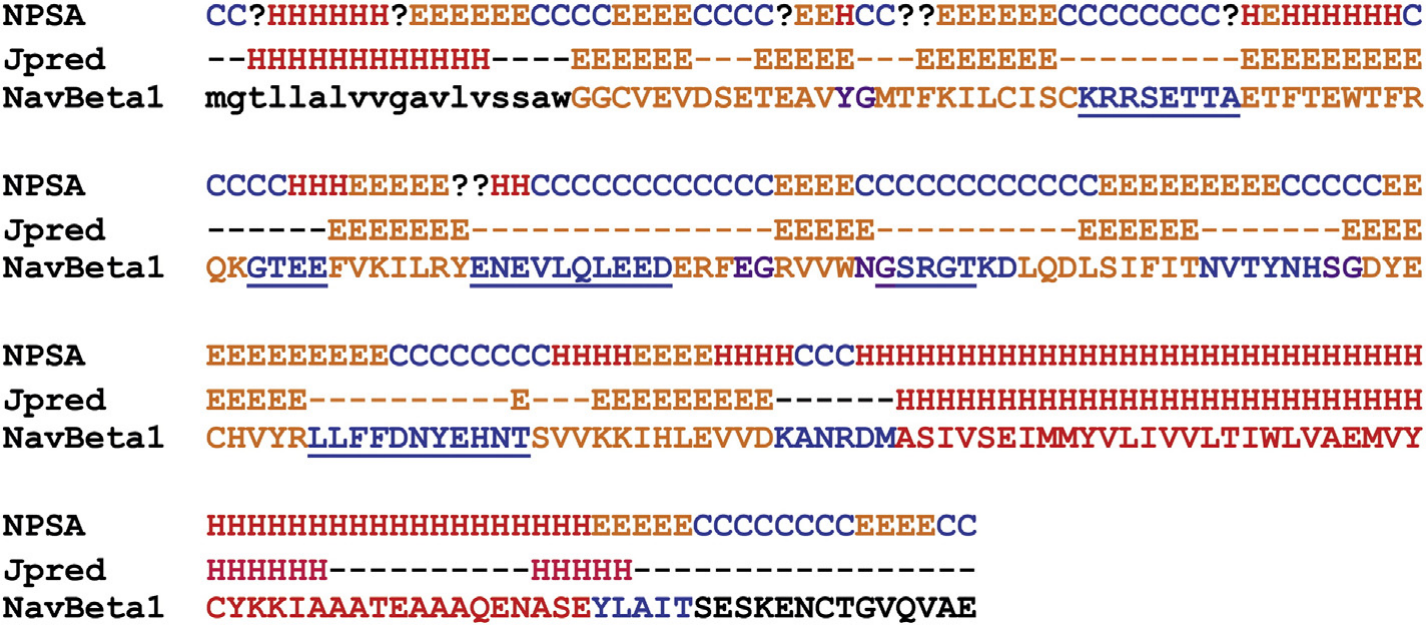
Consensus estimation for the secondary structure of the target Na_v_β1 amino acid sequence (third line) by NPSA (first line) and JPRED (second line) web tools [40,41]. Symbols: one letter coded amino acids, in red (helical, H), in brown (beta strands/sheet, E), in blue (loop/coiled C). The signal peptide (in positions1 to 18) was typed in lower case letters. For interpretation of the references to color in this figure legend, the reader is referred to the web version of this article.

After secondary structure prediction (Fig. 4) the primary sequence of Na_v_β1 was threaded through the aforementioned PDB templates, resulting in the empirical selection of our 3D template where the strands, loops and turns matched the predicted secondary structure of the target subunit.

With the secondary structure prediction at hand (Fig. 4) the appropriate 3D template was found in the chain X of 1HCF (PDB code: 1HCF_X [59]) which constitutes an “all β domain”, called d5 of the neurotrophic tyrosine kinase receptor type 2, known as cell surface receptor TrkB. As a most valuable asset d5 of TrkB showed a reversible interface with neurotrophin proteins (cf. literature for further details [59]) whereas higher scoring 1NEU [74] did not (Tables 2 and 3).

Furthermore, the immunoglobulins (antibodies, cf. literature for further details [60,61,72]) bind to antigens in a practically irreversible fashion (cf. antibody–antigen clumping in diagnostics).

**Fig. 5:**
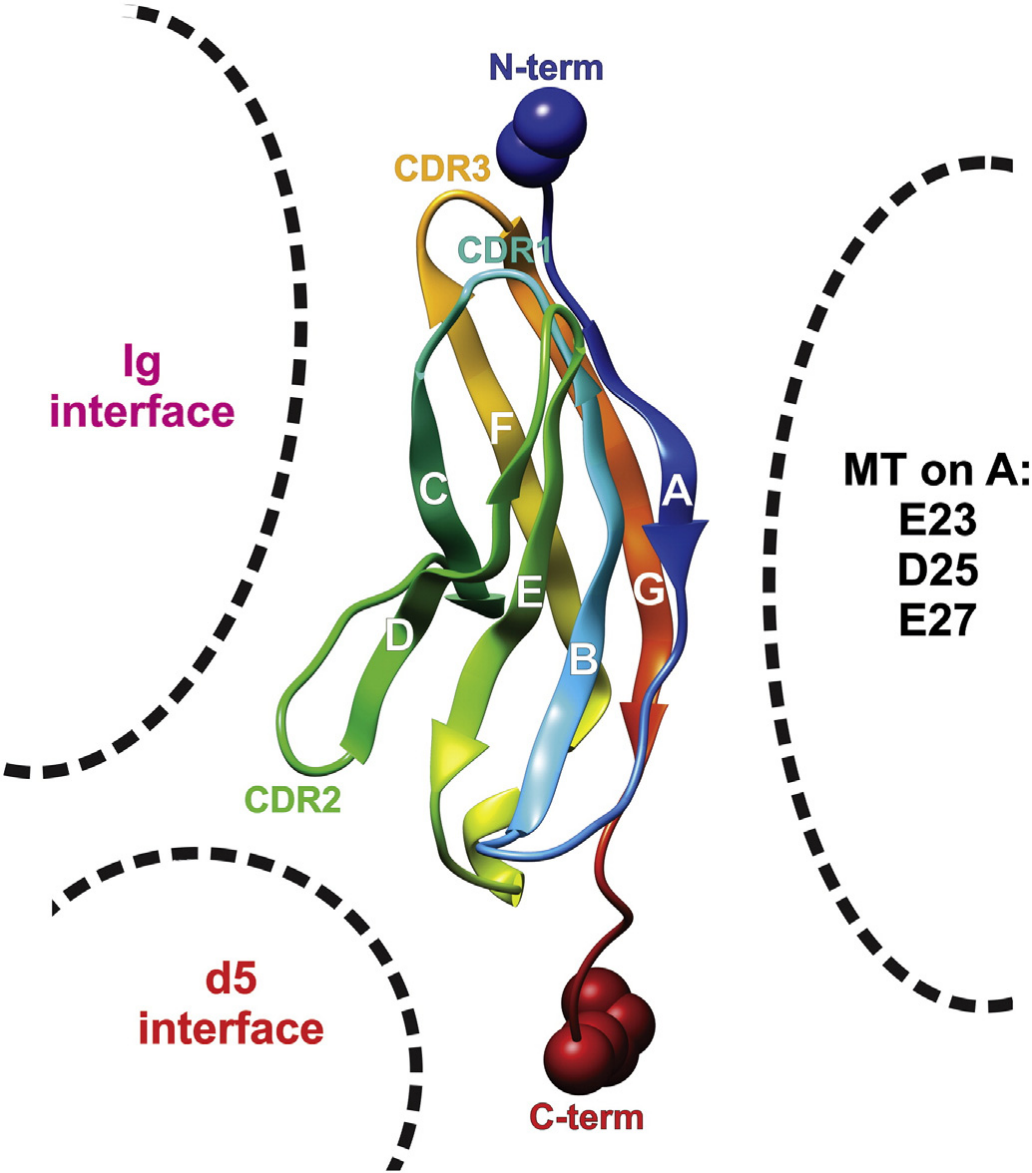
Once selected the 3D template, the known reversible and irreversible contact surface areas where localized by projecting the corresponding areas from superposed templates (Table 2, Fig. 3). According to the working hypothesis, only areas of reversible interfaces (d5) should be inspected as possible Na_v_1.4 α/β1 interface to determine residues for SDM studies. The literature [5,11] attests minor activities for mutant types (MT), e.g. glutamate 23 and 27 or aspartate 25 sitting on the C-term end (blue arrow point) of β strand A. CDR1, 2, 3 are the segments for irreversible association to antigens by antibodies (immunoglobulins, Ig). For interpretation of the references to color in this figure legend, the reader is referred to the web version of this article.

According to our ongoing electrophysiological study at that point in time it was hypothesized that Na_v_1.4 α and β1 subunits interact reversibly, all of which would be reflected by a Na_v_1.4 α/β1 interface with a reversible contact zone [11]. Subsequently, all target surface areas which correspond to the antigen binding sites of antibodies (CDR1, 2 and 3) could not interact with the channel's Na_v_1.4 α subunit. On the contrary, the β1 subunit in contact with the α subunit would rather correspond to a reversible interface like that seen in the TrkB complex [59]. In order to create “research exclusion zones” in the Na_v_1.4 α/β1 contact area under investigation, the CDR1, 2 and 3 regions of immunoglobulins were projected (by superposition) onto the 3D template [59] in addition to the mutated residues that only showed minor electrophysiological effects (Fig. 5) [5,11].

### 3.5 Step 5: the proof of concept: a multimeric protein complex with reversible and irreversible interfaces to a central “all β protein” (TLR4/MD-2 as the Rosetta Stone)

In the innate immune system, the Toll-like receptor (TLR) complex is situated on the cell surface to signal the presence (invasion) of smallest amounts of bacterial lipopolysaccharide (LPS) [76,77]. We used the crystal structure of the LPS-liganded human TLR4/MD-2 complex (PDB code: 3FXI [73]). The central myeloid differentiation factor 2 (MD-2) binds LPS as well as to two TLR4 proteins. It is a cell adhesion molecule. MD-2 folds into seven strands with a Greek-key motif building up two β sheets. Its shape resembles convex lenses but is open on one side to accommodate lipids. Moreover, MD-2 belongs to the Ig-like β-sandwich; E-set domain (early Ig-like fold family) is possibly related to the immunoglobulin family and implicated in lipid (LPS) recognition. It is attached to TLR4 and counter TLR4. The MD-2/TLR4 interface is very pronounced and the area of interaction enlarged during evolution. The MD-2/counter TLR4 interface adopts an enlarged area, though less pronounced. It is assumed that MD-2 associates to TLR4 permanently in contrast to the reversible association to the counter (second) TLR4 [73,76,77].

**Fig. 6:**
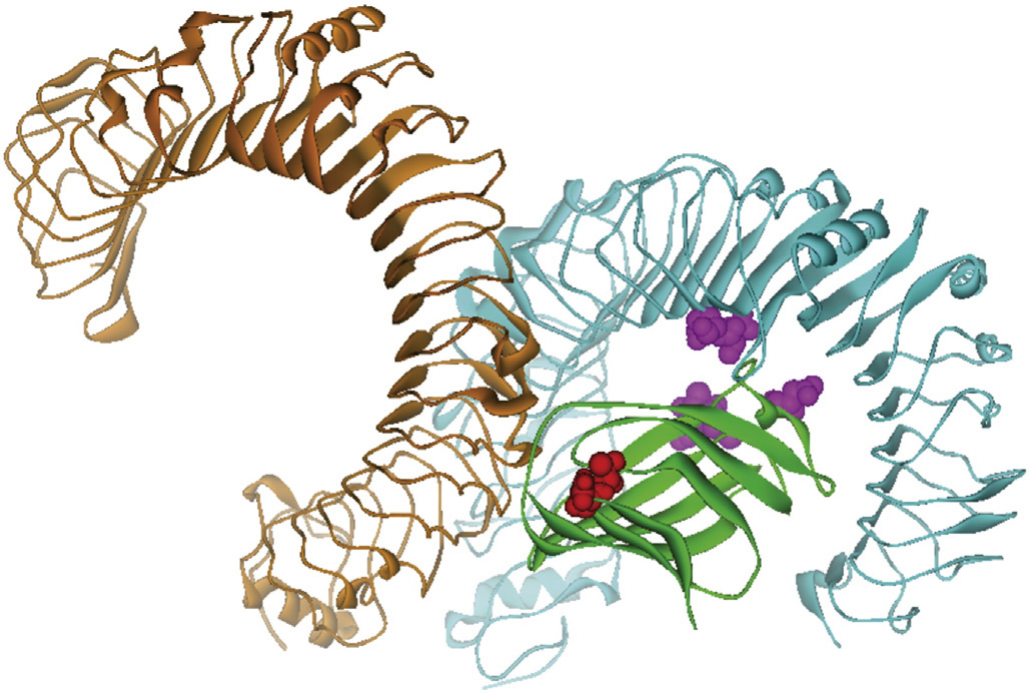
Main chains of TLR4 (blue), counter TLR4 (orange or brownish), all β protein MD-2 (green, equivalent Na_v_β1). All atoms are not displayed except for the irreversible Ig-like interface CDR1, 2, 3 regions (magenta) of an invisible, superimposed immunoglobulin and the reversible interface of 3D template 1HCF chain X [59]. Note: the template and Ig backbones of the 3D are omitted, as well as the counter MD-2 was redundant and not depicted. While the (blue) TLR4 is permanently bound to the MD-2, the counter TLR4 may leave upon antagonist binding into the MD-2 pocket [76,77]. This location coincides with the projected location of the reversible interface of neurotrophin-binding domain d5 of TrkB (red space-filling atoms) [59]. Note: CDR1, 2, 3 consist of a variable range of amino acids, but were formally represented by 1, 2 or 3 central residues, respectively (magenta colored space-filling atoms). For interpretation of the references to color in this figure legend, the reader is referred to the web version of this article.

Once in superposition onto MD-2 our Na_v_β1 target 3D model was inspected for potential protein–protein interaction areas on its surface in order to propose residues for mutation (Fig. 6). According to our prior studies [76,77] it has been cryptographically known that the TLR4/MD-2 complex binds reversibly a counter TLR4 (orange)/counter MD-2 (PDB code: 3FXI [73]). The evolutionary adaptation of enlarged interface areas (flaps) can also be observed in the case of MD-2 which permanently binds to TLR4 (cf. green and blue flaps flanked by the magenta space-filling atoms in Fig. 6) and to a lesser extent to the rightmost part of MD-2. In consequence, the only remaining area to look for potential zones of subunit–subunit interactions on Na_v_β1 is located towards the counter TLR4 (orange in Fig. 6) which becomes a “leaving group” when LPS antagonists bind into the MD-2 pocket(s) [73,78]. Two arguments prove our working hypothesis: (i) the irreversible interface between MD-2 (green) and TLR4 (blue) matches exactly the irreversible interface between an antigen and its antibody (magenta). (ii) The reversible interface between MD-2 (green) and counter TLR4 (orange) matches exactly the reversible interface between template d5 of Trk and its neurotrophin ligand (red). All told, the TLR4/MD-2 complex had the same relevance for us as the “Rosseta Stone” for French Egyptologist Champollion to decipher the hieroglyphs.

### 3.6 Step 6: generating the 3D model of the rat Na_v_β1 target subunit

**Fig. 7:**
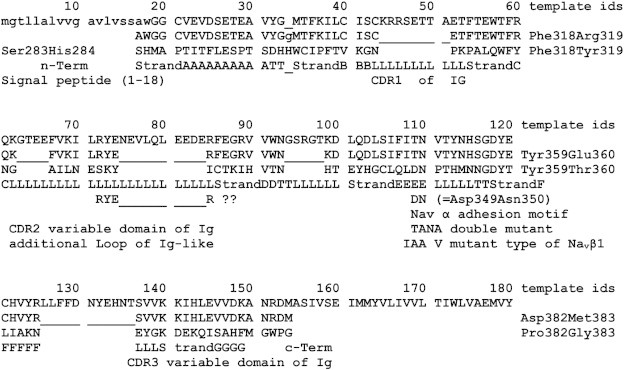
Manual construction of the target model after manual threading of the target sequence through the primary sequence of the 3D template (PDB code: 1HCF [59]). The final positions were achieved by accommodating beta strand and loop lengths and the *AnyGly* motif of type II turns [42]. The manual (not unattended) construction of the target 3D model of Na_v_β1 was achieved by SCWRL [44]. Line 1: aa (amino acid) count. The aligned blocks are flanked by residues (three-letter codes) with their respective id numbers as given in the 3D template, e.g. aligned E120 = template Glu360 [59]. Line 2: aa seq. of Na_v_β1 from Q00954 [4]. Line 3: aa seq. of Na_v_β1 manually threaded onto 1HCF chain X (Loops ___). The small capital “g” shows a glycine residue cut out. Then the local geometry was healed under the built-in GROMACs force field using SPDBV [37]. Line 4: aa seq. of TrkB d5 (3D template 1HCF_X [59]). Line 5: secondary structure: LLLL etc symbolizes; strandAAAA etc is the beta strands A; TT is a type 2 hair pin loop with a XG motif; “??” marks a doubtful hair pin XG motif. Line 6: hints about structures and functions of target and templates.

The 3D model of rat Na_v_β1 was generated using SCWRL 4 and Vega ZZ 3.0 [44,38]. At this stage – after the manual threading of the target sequence through the selected 3D template – the formation of type II hair pin loop turns (XG motif) and the Cys–Cys bridge was considered a key aspect to verify the residue positions (Fig. 7). Since the template complex showed a protein–protein interface between d5 of TrkB and a neurotrophin ligand (PDB Code: 1HCF [59] the target residues in the analogous α/β1 subunits interface should also bear typical side chains (interface forming Asp, Glu, Asn, Gln, Arg, Lys, His, Thr, Tyr; but deprecating Val, Leu, Iso, Ala, Pro, Met, Phe).

### 3.7 Step 7: identifying Na_v_β1 residues for mutational studies at the Na_v_ α/β1 interface

The reversible protein–protein contact zone between TrkB d5 and its neurotrophin ligand in the template complex indicated that surface area of Na_v_β1 is flanked by the irreversible CDR1, 2, 3 sites and the fruitless mutation points (Figs. 5 and 6). The template's physiological role is to trigger cell growth, differentiation and protection upon binding to tyrosine kinase receptors (Trk proteins) on the neural cell surfaces. Although the potential contact zone between both molecules (receptor: domain d5 of TrkB; ligand: neurotrophin) is far more extended, only a few side chains associate with noncovalent bonds which is in keeping with literature reports (Table 4) [80,81].

**Fig. 8:**
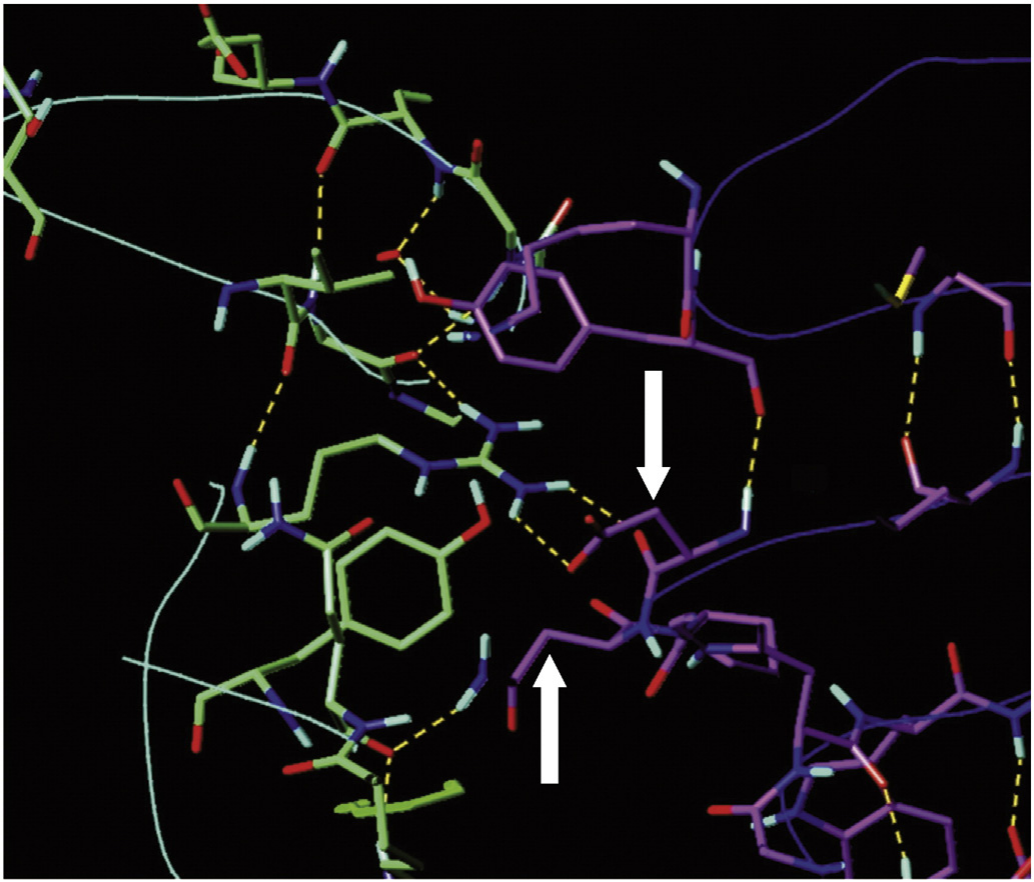
The three-dimensional model displays the amino acids on both sides of the ligand–receptor interface between neurotrophin (green) and receptor domain d5 of TrkB (purple) [59]. The hydrogen-bonding network is displayed (dashed yellow lines). Aspartic acid and adjacent asparagine (white arrows) mark the central part sitting on a loop turn (D349, N350). They were proposed to become the double mutant TANA (Fig. 7). Color code: carbon atoms of neurotrophin in green, O atoms in red, N atoms in blue, polar H atoms in light blue. All C atoms of binding domain d5 are held in purple color. The backbones of both proteins are displayed as lines. Nonpolar hydrogen atoms omitted for better viewing. For interpretation of the references to color in this figure legend, the reader is referred to the web version of this article.

Next, the spatial and chemical features at the template's reversible protein–protein interface were studied in details [79,81]. Historically, attempts to correlate mutation analysis to predict the 3D structure of proteins from the correlations in their aligned sequences fell short of expectations [80,82]. The reasons thereof are manifold. The explanations deviate from the scope and we refer to the literature instead [83,84,82]. Ligand binding and enzymatic activities are frequently located at protein and domain interfaces [85]. The analogue protein–protein complexes (Table 2) taught us nature's lesson that a typical noncovalent association of two distinct polypeptide chains does not exceed ten to twenty amino acids at all (Fig. 8) [79]. In addition, the template topology parallels that of the target α/β1 interface: of all five extracellular domains, the d5 domain is adjacent to the transmembrane helical segment in the primary sequence [86].

**Fig. 9:**
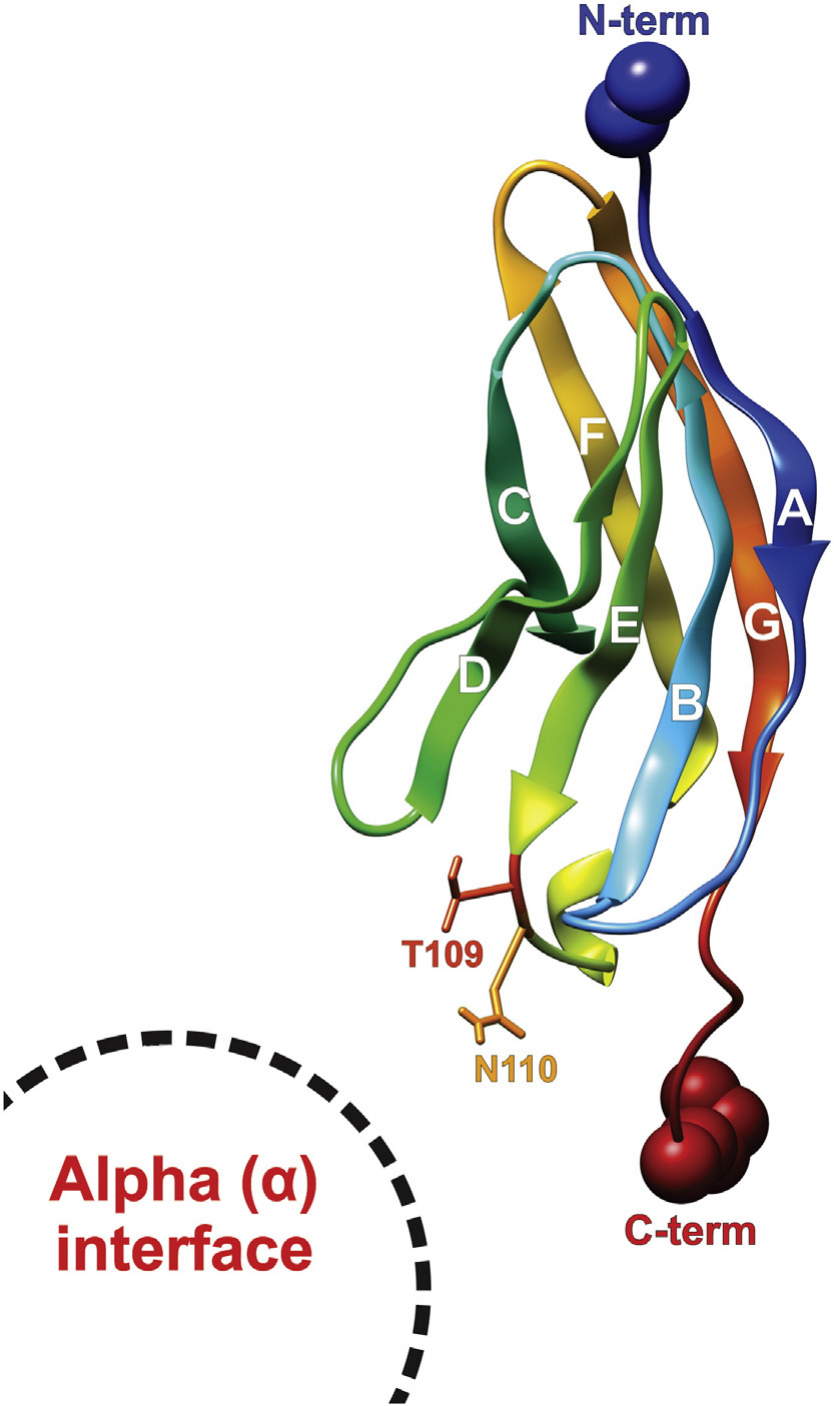
Display of the final Na_v_β1 3D model with the postulated interface with the α subunit. The location of the successful double mutant TANA becomes evident when comparing it to the template with the projected reversible and irreversible contact surface areas (Fig. 5). Each beta strand is labeled by its letter (from A to G). TANA (T109 → A N110 → A) lies on a prominent loop (flap) between strands E and F. The amino terminal side shows toward the extracellular space, while the carboxy terminal end of the ectodomain of Na_v_β1 is followed by the transmembrane and intracellular parts (Fig. 1). The protein backbone is displayed in rainbow colors from blue (N-term) over green, yellow and orange to red (C-term). Space-filling atoms mark the model endings. For interpretation of the references to color in this figure legend, the reader is referred to the web version of this article.

In good keeping with the literature attesting a protein binding role to prominent functional loop residues, the template's amino acids aspartate 349 and asparagine 350 were identified (Table 4, Fig. 8) and the corresponding target segment documented (cf. label “Asp349Asn350” in Fig. 7). Then both adjacent residues were mutated into alanine (T109A, NA) which gave the double mutant its name: TANA (Fig. 9). The combined SDM/electrophysiological studies with wild and mutant type TANA led to the long-awaited general loss of function of Na_v_1.4 channels in biological tests. Hence, the 3D model of Na_v_β1 successfully predicted two residues to disturb the interaction between the α/β1 subunit of the Na_v_1.4 channel.

### 3.8 Step 8: modulation of the inactivation of the voltage-gated sodium channel Na_v_1.4 by β_1_ subunit

**Fig. 10:**
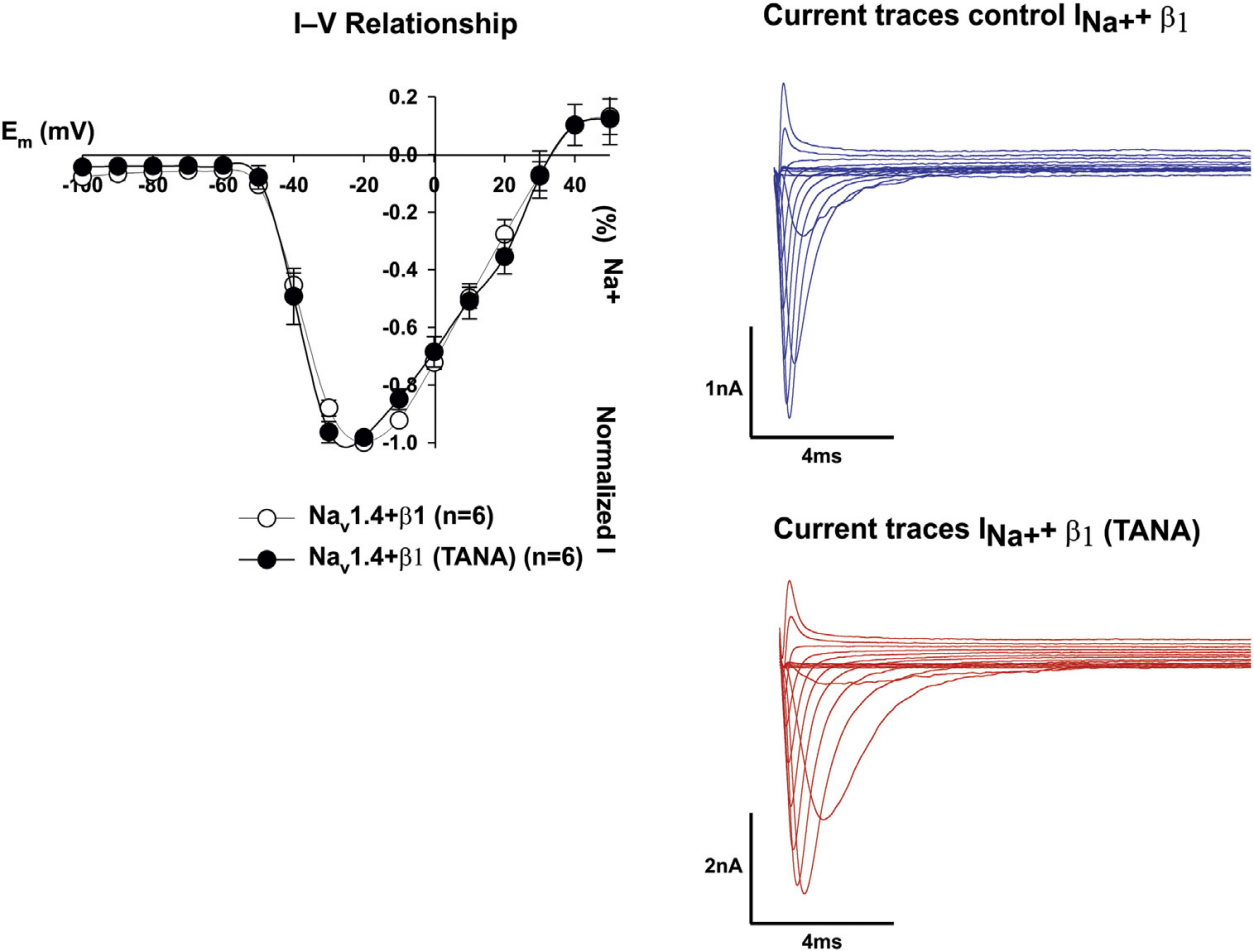
Representative current traces of the Na_v_1.4 sodium channel. Its α subunit was co-transfected with wild type (WT) β1 subunit (blue) or the double mutation TANA (red). Leftmost panel (black), the electrophysiological effects of a ß1 subunit mutation on the I-V relationship of Na_v_ 1.4. Sodium currents were generated by step depolarizations from a holding potential of − 100 mV in 10 mV increments from − 100 mV to + 50 mV (30 ms duration) in several mammalian CHO cells transfected with either WT α together with WT ß1 subunits as control or WT α subunit with TANA (red traces). Calibrations as shown, *n* = 6. For interpretation of the references to color in this figure legend, the reader is referred to the web version of this article.

**Fig. 11:**
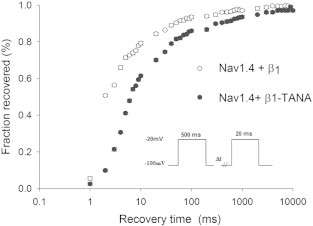
Recovery graph to compare wild type (WT: Na_v_1.4 and β1 subunit) and mutant type (MT: Na_v_1.4 and β1-TANA). Note, at time = 2 ms around (on the x axis) 50% of WT channels (0.5 on y axis) have recovered from inactivation, while only 10% of MT have recovered. After normalizing to percentage basis (100), WT = 100%, and MT is about 20%, yielding a difference of around 80%.

Voltage-gated Na^+^ channels formed by α and β subunits, characteristically display gating kinetics on millisecond time scales, ensuring rapid electrical communication between cells [2]. Na_v_β1 subunits interact non-covalently with pore-forming α subunits in the extracellular space, which accelerates gating kinetics, and modifies voltage dependence [2,4,8,87,88]. In this part of our study we observed that TANA disrupted inactivation, delayed recovery from inactivation and disrupted β1-like voltage-dependence (Fig. 10). TANA had neither an effect on the current–voltage (I–V) curve nor in the total amplitude of the current demonstrating that TANA did not change the peak voltage of activation (cf. leftmost inlay chart in Fig. 10). The observed loss of function can be classified as a general loss of function type because TANA had a double effect on: (i) kinetics of recovery from inactivation and (ii) high frequency stimulation. The general loss was about 80% by looking at the maximum difference between wild type (WT) and mutant type (MT) TANA (Fig. 11).

## 4 Discussion

The identification of the two aforementioned residues in the β1 subunit to interact with the α subunit was made possible based on a mixed homology and analogy approach exploiting hitherto unrelated topological and structural data of liganded proteins regardless of the degree of phylogenetic relatedness. In more explicit terms, the overall similarity percentage or identity score of MSA studies was not the driving force for decision taking. On the other hand the proposed mixed low-homology/analogy concept was not fool-proof either, on the contrary it required more personal expertise and user-attended modeling. The protein modeling was based on the secondary structure prediction and the proper identification of turns and loop segments. Myeloid differentiation factor 2 from our prior work with an “all β protein” complex helped us to distinguish between reversible and irreversible protein association interfaces. Supported by our electrophysiological data we postulated that the β1 subunit contacts α subunit in a reversible fashion. Then we identified two adjacent amino acids (T109, N110) because the corresponding residues (D349, N350) were also key binders in the reversible template complex (1HCF). After mutating both residues a general loss of sodium channel function was detected in our electrophysiology experiments.

## 5 Conclusions

The computed structure–function studies have resulted in the correct prediction of two adjacent functional residues which led to a loss of function in subsequent electrophysiological studies. Only a single attempt to identify two residues was necessary because the 3D model correctly pinpointed the subunit interface location. Our experimental results surpass previous electrophysiological attempts that have partially elucidated residues at the α/β1 interface. Our results contribute to the understanding of channel modulation and suggest a sequential interplay of both, α and β1 subunits, while the association of β1, through a distinct extracellular domain, accelerates gating.

## Figures and Tables

**Table 1:** Listing of inspected ion channel structures in search of suited 3D templates.

PDB code	Observations	Ref.
1T0J	Chains A and B: voltage-gated calcium channel subunit beta2a which is an intracellular domain and does not correspond to the ectodomain Na_v_β1. Chain C: voltage-dependent l-type calcium channel alpha-1c. Comparable to 4DEY.	[17]
4DEY	Chain A: voltage-dependent l-type calcium channel subunit beta. Chain B: voltage-dependent l-type calcium channel subunit alpha.	[50]
1ZSX	Chain A: voltage-gated potassium channel beta-2 subunit. It forms a heteromeric complex, without channel interface, albeit an analogous salt bridge exists (R74---E1114) between two regions with similarities to Na_v_β1.	[51]
3LUT	Chain A: voltage-gated potassium channel subunit beta-2. Chain B: potassium voltage-gated channel alpha subunit. The intracellular beta-2 segment has not an Ig-like fold. It has nothing in common with target Na_v_β1. The 499 residue-long alpha structure also presents the extra and intracellular loops. The latter have no homology with target loop regions.	[21]
2A79	Chain A: voltage-gated potassium channel beta-2 subunit. Chain B: potassium voltage-gated channel alpha subunit. Compare to newer 3LUT.	[52]
4EKW	Voltage-gated sodium channel in potentially inactivated state, from *Arcobacter butzleri* at 3.2 Å resolution, related to 3RVY, 3RVZ and 3RW0.	[12]
3RVY	Crystal structure of the voltage-gated sodium channel mutant Ile217Cys, at 2.7 resolution.	[13]
1QRQ	Mammalian beta subunit of K^+^ channels forming a structurally differing four-fold symmetric structure at 2.8 Å resolution, not related to target.	[19]
4L1D	Human sodium channel β3 subunit folds into an Ig domain, showing a homotrimeric complex in its crystal asymmetric unit.	[29]

**Table 2:** Listing of Ig-like templates with analogous interfaces. The percent identities of primary sequence against target amino acid sequence (NCBI Reference Sequence: NM_017288.1) are given for those templates which are discussed in more details (cf. asterisk).

PDB code	Observations	Ref.
1FHG	Telokin, C-terminus of smooth muscle myosin light chain kinase. It lacks the disulphide bridge between β-strands B and F.	[57]
1WWA	Tyrosine kinase receptor A, High affinity nerve growth factor receptor. Belongs to the I-set domain family of Immunoglobulins.	[53]
1WWB	Neurotrophic tyrosine kinase receptor type 2. Ligand binding domain of TrkB. Belongs to the I-set domain family of Immunoglobulins.	[53]
1WWC	Neurotrophic tyrosine kinase receptor type 3. NT3 binding domain of TrkC receptor. Belongs to the I-set domain family of Immunoglobulins.	[53]
1HCF	* (Brain-derived) Neurotrophic tyrosine kinase receptor type 2 in complex with neurotrophin-4 (NTF-4). Upon binding to NTF-4 TrkB undergoes homodimerization, autophosphorylation and activates. The receptor possesses an Ig-like β-sandwich fold; it belongs to the I-set domain family of Immunoglobulins, and the Ligand binding domain of TrkB. The neurotrophin has a cysteine-knot cytokines fold. (id score: 13%).	[59]
1JPS	* Chain i: light chain of immunoglobulin fab d3h44. Chain h: heavy chain of Immunoglobulin fab d3h44. The fab fragments embrace an Ig-like fold. The antibody shows an interface with an analogous flap of the tissue factor as its ligand (antigen). (id score: 18%)	[60]
3GRW	* Chain A: The ligand is the fibroblast growth factor receptor 3. Chain L: Fab light chain. Chain H: Fab heavy chain. The analogous interface is between the Ig-like fold domain of the antibody and FGFR3 comparable to 1JPS. (id score: 15%)	[61]
3KLD	* Chain A: contactin 4. Fragment: Ig-like domains 1-4. Chain B: tyrosine-protein phosphatase gamma. Fragment: carbonic anhydrase-like domain. The bound proteins form an analogous interface. (id score: 16%)	[62]
1HE7	Tyrosine kinase receptor A, High affinity Nerve Growth factor receptor. Belongs to the I-set domain family of immunoglobulins.	[69]
1HXM	T-cell receptor δ chain C region in complex with T-cell receptor γ-2 chain C region. They are heavy chains of immunoglobulins possessing C1-set (constant) and V-set (variable) domains. They have an Ig-like β-sandwich fold.	[70]
1BD2	T cell receptor complex, formed by an HLA class I histocompatibility antigen, A-2 α chain, a leukemia viral peptide and an alpha-beta T cell receptor (TCR), B7. Two human T cell receptors bind in a similar diagonal mode to the HLA-A2/Tax peptide complex using different TCR amino acids.	[71]
1KXQ	* Camelid heavy chain variable domain (Vhh) antibody in complex with porcine pancreatic α-amylase. (id score: 19%)	[72]
3D2F	Chains A and C: Heat shock proteins complex (Hsp). Nucleotide exchange factor (NEF) Sse1p of Hsp110 bound to nucleotide-binding domain (NBD) of Hsp70. The yeast Sse1p Hsp110 possess a 2 layer (bred buns) sandwich architecture.	[58]
3FXI	* Toll-like receptor (TLR4) in complex with myeloid differentiation factor 2 (MD-2) and a bacterial lipopolysaccharide (LPS). The Toll-like receptor has a leucine-rich repeats (LRR), flanked by cysteine-rich domains common in cell adhesion molecules among other proteins. (id score: 11%)	[73]
1NEU	* Chain A: extracellular domain from the major structural protein of peripheral nerve myelin with a typical Ig-like fold; five residues at the C-terminus are disordered, suggesting a flexible linkage to the membrane. (id score: 23%)	[74]

**Table 3:** Multiple sequence alignment by Clustal W [39]. Homology between template (rBeta1) and 3D templates was very low. Na_v_β3 (4L1D) and Na_v_β4 (4MZ2) were not available at the time of modeling. Homology was expressed as percentage identity score between the aligned sequences. The threshold below which the twilight zone (uncertainty) for homology modeling exists was estimated according to literature (Fig. 14.4, in [75]).

PDB code	Residue length	Overall id score	Threshold (approx.)	Twilight Zone	Ref [4]
rBeta1	218	100%			
4L1D_A	127	45%	30%	No	[29]
4MZ2_A	129	20%	30%	Yes	[30]
1HCF_X	101	19%	30%	Yes	[59]
3KLD_A	384	18%	20%	borderline	[62]
1NEU_A	124	27%	28%	borderline	[74]
3FXI_C	140	11%	25%	Yes	[73]
3GRW_A	241	15%	22%	Yes	[61]
1KXQ_E	120	19%	28%	Yes	[72]
1JPS_H	225	16%	23%	Yes	[60]

**Table 4:** Listing of the observed nonbonded interactions between the ectodomain (d5 or Ig2) of TrkB-d5 and its neurotrophin-4/5 ligand (Fig. 8).

Protein interface	Ligand residues	Receptor residues
Observed nonbonded interactions for columns 2 and 3	Two observed complexes with NGF/NT4/5 [59,69]	NT-binding domain d5 of NTR: TrkA/B/C [59]
No/No	No/No	T325/S327/S345
No/No	No/No	S326/K328/K346
No/npHb/Weak pHb	No/No E35/E37 + R114	F327/Y329/I347
+−/+−	R103/R114	N349/**D349**/N366
Hb/Hb	H84/Q94	Q350/**N350**/K367
wHb/Hb	H2O/E13	H297/H299/R316* (* not adjacent L315 or H317)
np/Hb	I6/R10 (bb)	L333/H335/Y353* (* not adjacent Y352, H349)

The amino acids of the analogous protein–protein interface to the Na_v_ α/β1 interaction site are represented with their one-letter codes. The analogy data were retrieved from crystal structures (PDB codes: 1WWW [53,86] and 1HCF [59]). Legend of symbols: (w or p)Hb = (water-mediated or polar hydrogen bonds; (+-) = salt bridge; (no) = not observed; (np) = nonpolar or hydrophobic; (bb) = protein backbone or main chain. The two residues in bold face (D349, N350) correspond to T109, N110 of TANA.
